# A Systematic Review of Behavioral, Physiological, and Neurobiological Cognitive Regulation Alterations in Obsessive-Compulsive Disorder

**DOI:** 10.3390/brainsci10110797

**Published:** 2020-10-29

**Authors:** Sónia Ferreira, José Miguel Pêgo, Pedro Morgado

**Affiliations:** 1 Life and Health Sciences Research Institute (ICVS), School of Medicine, University of Minho, 4710-057 Braga, Portugal; soniamgaf@gmail.com (S.F.); jmpego@med.uminho.pt (J.M.P.); 2 ICVS-3B’s PT Government Associate Laboratory, 4710-057 Braga/Guimarães, Portugal; 3 Clinical Academic Center-Braga (2CA), 4710-243 Braga, Portugal

**Keywords:** OCD, obsessive-compulsive, emotion, suppression, reappraisal, fMRI

## Abstract

Obsessive-compulsive disorder (OCD) is characterized by cognitive regulation deficits. However, the current literature has focused on executive functioning and emotional response impairments in this disorder. Herein, we conducted a systematic review of studies assessing the behavioral, physiological, and neurobiological alterations in cognitive regulation in obsessive-compulsive patients using the PubMed database. Most of the studies included explored behavioral (distress, arousal, and frequency of intrusive thoughts) and neurobiological measures (brain activity and functional connectivity) using affective cognitive regulation paradigms. Our results pointed to the advantageous use of reappraisal and acceptance strategies in contrast to suppression to reduce distress and frequency of intrusive thoughts. Moreover, we observed alterations in frontoparietal network activity during cognitive regulation. Our conclusions are limited by the inclusion of underpowered studies with treated patients. Nonetheless, our findings support the OCD impairments in cognitive regulation of emotion and might help to improve current guidelines for cognitive therapy.

## 1. Introduction

Obsessive-compulsive disorder (OCD) is characterized by recurrent intrusive thoughts (obsessions) and repetitive or ritualistic actions or mental acts intended to diminish the anxiety and distress elicited by obsessions (compulsions). OCD patients often have comorbid conditions including anxiety disorders, major depressive disorder, bipolar disorder, obsessive-compulsive personality disorder, and tic disorders [1]. In addition to its distinctive symptoms, OCD is defined by cognitive deficits involving memory and attentional biases towards relevant/threatening stimuli, memory distrust, and difficulty in accessing internal states. Thus, these patients depend on external stimuli and reassurance [2,3]. The past literature has focused on the study of executive function in OCD patients, mainly by using memory, inhibition, attentional shifting, reversal learning, and interference tasks [3,4,5]. Given that OCD patients might be frequently focused on controlling or responding to their obsessions, they might have an overall impaired performance on executive tasks. They might have reduced cognitive flexibility during task performance because their cognitive resources are engaged by obsessive thoughts [4,5,6,7]. Indeed, prior research showed evidence that cognitive flexibility deficits emerge in emotionally relevant contexts for OCD patients (activation of disorder-specific fears), supporting the association between cognitive inflexibility and obsessive symptoms [8].

Early cognitive and behavioral models of OCD (Salkovskis [9], Rachman [10], and Obsessive Compulsive Cognitions Working Group [11]) proposed that obsessions and compulsions result from cognitive deficits in the interpretation of thoughts [9,10,11]. OCD patients have dysfunctional beliefs of higher significance/need for control of thoughts, inflated sense of responsibility, perfectionism, intolerance of uncertainty, and overestimation of threat [2,12,13,14,15]. Despite the augmented necessity to control thoughts, OCD individuals apply suboptimal strategies that intensify their occurrence: compulsions, neutralizing, suppression, and worry [2,12,13,14,16,17].

Cognitive regulation consists of the pliable modulation of cognition arbitrated by central and peripheral systems [18,19]. This regulation involves top-down/deliberate and bottom-up/automatic mechanisms. Bottom-up mechanisms are associated with automatic responses to external/sensory stimuli in subcortical regions (amygdala and ventral striatum/ventral tegmental area). Top-down processes respond to internal mental representations (e.g., goals/rules) and include brain responses in the anterior cingulate cortex (ACC) and the ventromedial, ventrolateral (vlPFC), and dorsolateral prefrontal (dlPFC) cortices to modulate the activity in subcortical regions [6,18]. The cingulo-opercular (vlPFC, dorsal ACC, and anterior insula) and frontoparietal (dlPFC, posterior/inferior parietal and inferior temporal cortices) networks are associated with cognitive regulation [18,19]. The frontoparietal network is responsible for the allocation of attention, while the cingulo-opercular network adjusts goal-related information and processes salient stimuli [19]. These networks interact through connections with the thalamus, hippocampus, and cerebellum [18,19]. The cingulo-opercular network mediates the correlation between the frontoparietal and default-mode networks during rest and cognitive control tasks [19].

Previous research indicates the existence of cognitive regulation impairments in OCD. However, to the best of our knowledge, no attempts have been made to review prior findings. Previous authors have focused on reviewing executive functioning and emotion processing in OCD [3,4,5,20]. Herein, we systematically reviewed the past literature to elucidate the main cognitive regulation processes impacted by OCD. We focused on studies assessing objective behavioral, physiological, and neurobiological parameters and not subjective self-reported data such as psychometric scales [21]. In this way, this work might contribute to the understanding of cognitive regulation mechanisms altered by OCD, including peripheral and central nervous system responses and behavioral manifestations/changes during cognitive regulation. Our review can enlighten the current application of therapies based on cognitive regulation strategies and the development of new treatment approaches combining physiological/neural signals and cognitive regulation strategies (e.g., biofeedback and neurofeedback).

## 2. Materials and Methods

We followed the Preferred Reporting Items for Systematic Reviews and Meta-Analyses (PRISMA) norms [22,23] for the systematic review. We searched PubMed (Medline) database on the 14 April 2020 using the combination of the following terms: (OCD OR “obsessive-compulsive disorder” OR “obsessive compulsive disorder”) AND (regulation OR reappraisal OR control) AND cognitive. We restricted the findings to articles in English, with human participants, with the availability of a full-text document, and reporting original results (reviews and book chapters were excluded). The author SF conducted the search and the eligibility assessment. The results were discussed among all authors in case of doubt. First, we selected the articles by the title and then by the abstract content. Later, the full text of the articles was analyzed according to the inclusion criteria. The inclusion criteria were: (1) the existence of a control group with non-psychiatric participants (controls); (2) the existence of a patients’ group with a primary diagnosis of OCD based on validated instruments (e.g., Diagnostic and Statistical Manual of Mental Disorders (DSM) [1]); (3) the inclusion of a direct statistical comparison between the control and the OCD group; (4) the assessment of cognitive regulation with behavioral, physiological, or neurobiological measurements. The exclusion criterion was the sole use of self-reported measures of cognitive regulation (e.g., psychometric scales or questionnaires). Both cross-sectional case-control and interventional controlled studies were included.

We extracted the following information from each article: (1) group characterization; (2) group size; (3) group mean age; (4) group gender ratio; (5) diagnosis instrument; (6) mean Yale-Brown Obsessive Compulsive Scale (Y-BOCS) total score; (7) treatment approaches; (8) psychometric characterization related to cognitive regulation; (9) task description; (10) behavioral results; (11) physiological outcomes; (12) neurobiological findings; (13) techniques employed. Studies with common authors were carefully analyzed to avoid data duplication.

## 3. Results

**Figure 1:**
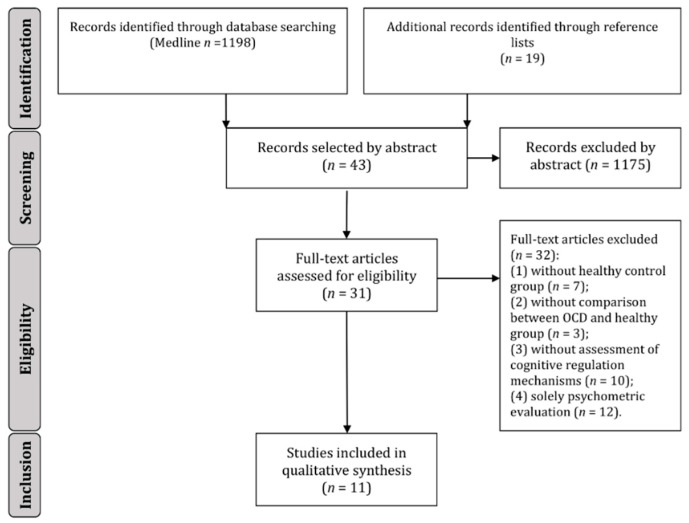
Flow diagram of the literature search (adapted from [22,23]).

Figure 1 represents the selection process. The search yielded a total of 1198 studies and 19 articles were additionally identified through reference lists. No unpublished studies were found in the reference lists of the included studies. After abstract reading, we selected 43 articles and we included 11 studies after full-text reading. Two studies used the same sample [24,25] and one study had two experiments with distinct samples [26] (one with an overlapping sample from another study [27]). Thirty-two reports did not meet the study criteria: 12 articles only assessed self-reported measures of cognitive regulation; 10 articles did not explore cognitive regulation processes; 7 reports did not incorporate a healthy control group; 3 studies did not statistically compare OCD and control participants. The final selected articles were published between 1999 and 2019 by authors from the USA [26,27,28,29], Germany [30,31,32], The Netherlands/Sweden [24], The Netherlands/Norway [25], Turkey [33], and Spain/South Africa/USA [34].

The studies included 301 OCD patients and 254 healthy participants in total, with an average of 27.4 ± 17.6 patients (mean ± standard deviation) and 23.1 ± 11.2 control participants per study. The average age for OCD participants was 31.5 ± 3.8 years and 30.8 ± 5.1 years for controls. On average, 49.7 ± 8.1% of OCD patients and 54.4 ± 9.3% of controls were female. This agrees with past literature demonstrating that OCD has an equal prevalence for both genders in adulthood [35]. All OCD patients were diagnosed with the DSM-IV and had an average Y-BOCS total score of 22.4 ± 1.3 (one study with missing information). Five articles explored behavioral tasks (Table 1) and six studies evaluated neurobiological and/or behavioral processes with functional magnetic resonance imaging (fMRI) and electroencephalography (Table 2).

The selected studies comprised mostly the cognitive regulation of thoughts, mental images, or pictures. The authors evaluated the distress, disgust, arousal, and frequency of thoughts as the main behavioral outcomes, and brain activity/functional connectivity as neurobiological parameters. The tasks required the use of the following cognitive regulation strategies: suppression, distraction, acceptance, rescripting, and reappraisal.

The studies found that the suppression of negative/intrusive thoughts leads to an increase in the frequency of these thoughts during and after suppression [28,29], and an augmentation of the distress elicited by the thoughts after suppression [29] in OCD participants. Other authors found that the suppression of neutral thoughts (e.g., thinking about a “white bear”) results in increased frequency of the target thought for OCD individuals solely during suppression [26,27]. Moreover, Koçak and colleagues reported better performance for the OCD group during the suppression of an abstract mental image [33]. Najmi et al. [29] demonstrated that the distress associated with intrusive thoughts was higher after using suppression when compared to distraction and acceptance strategies. Additionally, they demonstrated that intrusive thoughts were more frequent after the suppression in comparison to the acceptance condition in OCD patients. Lastly, they reported that the distress caused by intrusive thoughts diminished after applying acceptance strategies in OCD individuals [29]. Other authors reported a reduction of arousal for aversive pictures after using reappraisal compared to distraction techniques in OCD individuals [31], and a decrease in distress for OCD-related pictures during the reappraisal condition [24,25]. Fink and colleagues [30] also found decreased disgust ratings for OCD-related pictures after cognitive reappraisal in OCD and control participants, but no statistically significant differences between groups.

**Figure 2:**
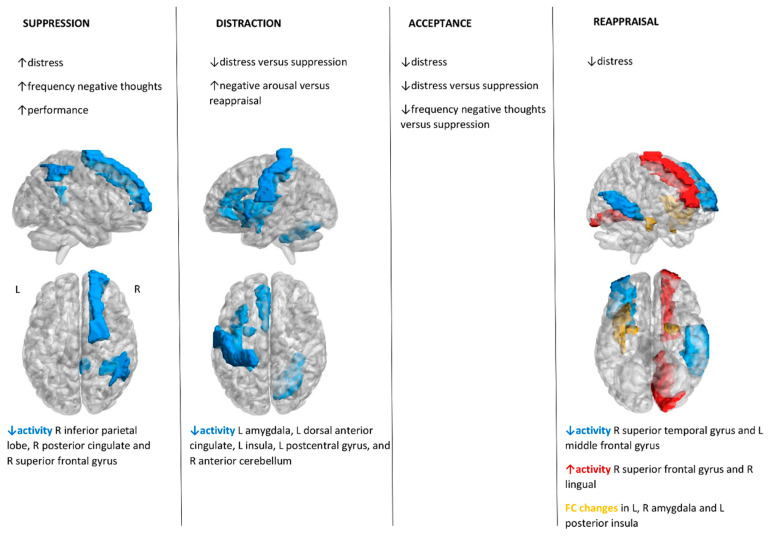
Summary of the systematic review of behavioral and neurobiological findings for the obsessive-compulsive versus the healthy control groups. R—right; L—left. The brain maps were created with the BrainNet Viewer using the Automated Anatomical Labelling atlas.

Cognitive reappraisal of fear-related pictures corresponded to decreased activity in the left middle frontal gyrus and right superior temporal gyrus, while reappraisal of OCD-related pictures increased activity in the right superior frontal gyrus and right lingual gyrus for OCD patients [24,25]. Moreover, these authors reported decreased functional connectivity in the left posterior insula and right amygdala during the reappraisal of fear-related images [24,25]. These results were consistent with the altered correlation between functional connectivity of the left amygdala–left posterior insula and the reappraisal abilities in OCD individuals reported by other authors [34]. Distraction strategies during the presentation of OCD-related pictures led to decreased activity in a left cluster including the amygdala, dorsal ACC, insula, and postcentral gyrus, and the right anterior cerebellum in OCD participants [32]. Both reappraisal and distraction strategies during the visualization of pictures were associated with decreased responses in centro-parietal regions (late positive potential) in healthy participants but not in OCD patients [31]. Lastly, suppression and manipulation of a mental image were linked to decreased activity in the right hemisphere in the inferior parietal lobule, posterior cingulate cortex, and superior frontal gyrus in OCD [33]. Figure 2 contains a summary of these findings and the Supplementary Table S1 presents a detailed description of the studies’ behavioral tasks.

All the studies included were cross-sectional case-control studies. We assessed the quality of the studies with the National Institute for Clinical Excellence (NICE) checklist for case-control studies [36]. Overall, the selected publications had good quality in terms of the definition of the research question, exclusion criteria for cases and controls, and differentiation between cases and controls. Some studies failed to recruit comparable groups for cases and controls, to clearly address confound variables, and to describe statistical results with error values. None of the studies reported information about the participation rate and differences between participants and non-participants (Table 3).

## 4. Discussion

Herein, we systematically reviewed studies assessing cognitive regulation alterations in OCD in terms of behavioral, physiological, and neurobiological findings. Concerning behavior, these studies showed that distress and intrusive thoughts can be attenuated by cognitive reappraisal and acceptance strategies. Neuroimaging studies found altered brain responses mainly in dorsal prefrontal, temporal, parietal, and limbic regions during cognitive regulation in OCD participants. Other authors showed that reappraisal capacity was associated with functional connectivity changes between the amygdala and posterior insula in OCD.

Psychiatric diseases are generally characterized by impaired emotion regulation abilities, with excessive suppression and reduced acceptance of emotions [37,38]. Previous literature using psychometric instruments demonstrated that OCD patients have difficulties in cognitive regulation. They reported increased deficits in emotion regulation, namely diminished reappraisal abilities and increased use of suppression strategies [39,40]. Some of the studies included in this review also indicated the same trend by using self-reported questionnaires [24,25,30,31,34]. The beneficial effect of reappraisal over suppression in OCD patients and other individuals has been supported by past findings [21,41,42]. These authors denoted that reappraisal occurs before the complete unfolding of the emotional response and is more effective to control the negative impact of emotions, while the suppression process starts during the emotional response itself [21,41,42,43,44].

Suppression consists of the inhibition of emotions, physiological responses, or behaviors in the face of stimuli [41,42,43]. Thought suppression might become chronic if associated with unpleasant emotions and lead to increased frequency of suppressed thoughts [29,44]. Our findings support the notion that suppression is a maladaptive strategy in OCD because it is linked to a subsequent higher occurrence of intrusive thoughts and enhanced distress [28,29]. Our results also showed that suppression is linked to increased internal attributions of weakness and incapacity to control intrusive thoughts in OCD [27]. Thus, OCD patients might often adopt suppression strategies as an effort to control obsessions [29]. Indeed, one of the studies included in this review showed that OCD patients have a higher performance during suppression [33]. In contrast to previous findings demonstrating increased responses in the dlPFC and inferior parietal cortex during suppression in healthy individuals [42], Koçak et al. (2011) found blunted superior frontal gyrus and inferior parietal lobule activity in OCD during suppression. The inferior parietal cortex is involved in shifting attention away from the self [42]. The dlPFC involvement in cognitive regulation is discussed below. These altered responses in prefrontal and parietal cortices might underline the maladaptive use of suppression in OCD.

Distraction consists of shifting attention away from intrusive thoughts to focus on neutral/alternative stimulus [21,29,42,45]. In this review, we observed that distraction reduces the distress elicited by intrusive thoughts when compared to suppression [29]. Moreover, other authors indicated decreased responses in the amygdala, dorsal ACC, insula, postcentral gyrus, and cerebellum in OCD participants during distraction [32]. Previous studies demonstrated increased responses in the ACC and parietal cortex and diminished activity in the amygdala and insula using distraction paradigms in healthy individuals [42,45]. The dorsal ACC and the inferior/superior parietal cortex are responsible for the allocation of attentional resources [38,42] and present decreased activity during reappraisal in individuals with anxiety disorders [38]. The dorsal ACC is also associated with the update of working memory and performance monitoring [42] and provides the connection between areas involved in the appraisal of affective stimuli (e.g., amygdala) and vlPFC and dlPFC regions associated with the initiation and execution of regulation [46]. Thus, despite the indication of reduced distress and amygdala and insular activity with the use of distraction strategies, OCD patients might have functional impairment in ACC and parietal areas. Lastly, distraction seems to be effective in the short term to decrease stress and negative arousal but not as a recurrent emotional regulation strategy, mainly when compared to reappraisal strategies [21,42,44], as reported by Paul and colleagues [31].

Acceptance refers to the experience of distressing situations without trying to alter their meaning [29,47]. Mindfulness techniques involve acceptance, consisting of nonjudgmental awareness of an experience [21,48]. Individuals with higher distress tolerance have stronger acceptance and mindfulness skills [44]. Thus, acceptance-based strategies might be efficient to target the distress elicited by obsessions. Indeed, previous studies showed that the acceptance and commitment therapy has beneficial effects for OCD patients, namely in reducing the severity of obsessive, compulsive, anxiety, and depressive symptoms [49,50]. Moreover, the recurrent employment of acceptance is associated with reduced use of maladaptive strategies such as suppression and decreased level of depressive and anxiety symptoms [21,47]. Thus, acceptance-based strategies might be adopted to treat OCD patients when standard cognitive-behavioral therapy (CBT) is unavailable or as a complement to CBT.

Cognitive reappraisal involves the modification of the significance of initial appraisals [38,42,43,46,51,52]. The most common reappraisal strategies are the reinterpretation of the stimuli with a more positive meaning or distancing from it with the viewing perspective of an unrelated observer [21,37,38,41,42,45,48,51]. In line with our conclusions, the previous literature points to increased activity in the inferior frontal gyrus, posterior insula, and occipitotemporal regions, and decreased response in the dorsomedial prefrontal cortex (dmPFC)/dlPFC and temporal gyrus during cognitive reappraisal in patients with mood and anxiety disorders [37,38]. Moreover, studies with healthy individuals also reported the involvement of the dlPFC/dmPFC, parietal and temporal cortex, and the amygdala and insula in cognitive reappraisal processes [42,45,51,52]. The prefrontal alterations might indicate the deficient allocation of attention and impaired monitoring/manipulation of emotion-related information [37,38,42,51,52]. The increased activation of occipitotemporal regions might translate into enhanced attention to negative stimuli [37,52]. Additionally, the prefrontal and parietal cortex have a modulatory effect on lateral temporal regions associated with semantic and perceptual representations to alter the emotional significance of external stimuli [42,52]. In line with the findings of Paul et al. [31] reviewed here, the downregulation of emotions during reappraisal is also linked to decreased late positive potential amplitude in centroparietal regions, representing a reduction in sustained attention towards the negative stimuli [38]. Moreover, patients with anxiety disorders have decreased inferior/superior parietal responses during reappraisal of negative stimuli [38] that might be associated with impaired inhibitory control [37] or blunted recruitment of attentional resources [38]. Thus, OCD patients seem to have impaired cognitive reappraisal processes because they did not present diminished late positive potential. Previous studies also found that healthy participants with higher reappraisal abilities have lower values of functional connectivity between the amygdala and anterior insula [43] and that the anterior insula activity is associated with the amygdala function during emotion regulation [46]. Additionally, the posterior insula and amygdala responses are downregulated by reappraisal strategies [42]. These authors suggest that the insula is involved in the selection of appropriate strategies to subsequently downregulate the amygdala activity in the face of negative emotions. The absence of this association in OCD individuals [34] might indicate that their cognitive reappraisal deficits are underlined by an impaired functional connection between amygdalar and insular regions. The results from other authors included in our review also support impairments in functional connectivity in the amygdala and insula during fear reappraisal [24,25].

CBT is one of the first-line treatments for OCD [53,54] and aims at improving negative appraisals and dysfunctional beliefs with reappraisal strategies [44,52,55,56]. After CBT, the activity in brain regions associated with affective processing (orbitofrontal cortex, ACC, thalamus, and caudate) usually decreases and there is an enhancement of brain response in regions linked to neurocognitive processes (dlPFC, parietal cortex, putamen, and cerebellum) [55,57]. However, some studies also report the reduction of dlPFC activity after CBT [55]. Thus, CBT seems to restore prefrontal control over subcortical regions [55] by increasing activity in frontal and parietal regions. Indeed, improved set-shifting, inhibitory, visuospatial, verbal memory, and working memory abilities have been reported after CBT and cognitive training [58]. Moreover, dysfunctional beliefs decrease after CBT and cognitive therapy [56,59,60], although other authors found controversial results [61,62].

Our conclusions are limited by the modest sample sizes (more than half of the studies with less than 30 participants per group), the concurrent medication and/or CBT (only three studies included patients without medication [24,25,32]), and the inclusion of patients with comorbidities (e.g., major depressive disorder, anxiety disorders, and phobias; only one study reported the exclusion of comorbidities [33]). Additionally, many of the studies did not provide information about the different OCD dimensions of the patients included, hindering the comparison between patients with different symptomatology. Moreover, the majority of the publications did not study correlations between the main outcome measures and the severity of OCD disorder. Most of the studies also lacked information about specific treatment approaches and treatment duration, as well as the age of onset of OCD, preventing the analysis of putative effects of these factors on the main outcomes. Moreover, the majority of the studies explored suppression and reappraisal strategies, preventing the extraction of robust information for other strategies (e.g., acceptance). Most of the studies employed emotion-related stimuli or paradigms analyzing intrusive thoughts (except [33]). OCD is also characterized by an imbalance between cognitive and reward pathways that explains the execution of rewarding compulsive actions in response to uncontrollable obsessional thoughts [63]. Thus, tasks of cognitive regulation of reward processing are critical for future studies [64]. Lastly, some studies might have not been included in this review because our search process was conducted only in the Medline database. However, we complemented our search with reference lists.

To better tackle the cognitive regulation alterations in OCD, future studies should use cognitive regulation tasks assessing behavioral parameters (e.g., distress, anxiety, and occurrence of intrusive thoughts) in combination with neuroimaging methods with the additional incorporation of physiological measures (e.g., heart and respiratory rate and skin conductance) to obtain objective parameters of anxiety/distress changes. Moreover, the inclusion of treatment naïve patients and the use of larger samples is crucial. Additionally, the use of more ecological/personalized approaches might be more appropriate to disentangle the mechanisms involved (e.g., asking OCD participants to regulate their obsessions without using other external stimuli) [21].

## 5. Conclusions

This review provides further insight into the cognitive regulation alterations in OCD that might guide the improvement of cognitive therapy and CBT. Overall, we observed altered brain responses in regions belonging to the frontoparietal network (dlPFC/dmPFC, inferior/superior parietal cortex, and superior temporal cortex) during cognitive regulation. This conclusion suggests an impairment in attention allocation and deficient control of emotion-related information [18,19]. Moreover, this review suggests a beneficial effect of reappraisal and acceptance strategies and a detrimental effect of suppression approaches regarding the reduction of distress and frequency of intrusive thoughts after cognitive regulation. Nonetheless, further research needs to be conducted to compare the efficacy of different cognitive regulation strategies. The studies included in this review described experiments involving suppression, distraction, acceptance, and cognitive reappraisal strategies. In this way, this review highlights the lack of studies exploring the effects of other cognitive regulation strategies for OCD patients (e.g., neutralizing and worry).

## Figures and Tables

**Table 1:** Summary of the studies with behavioral measures of cognitive regulation. The psychometrics and results comprise direct statistical comparisons between groups (OCD vs. healthy).

Study	Groups	Size	Age (years)	Gender (%F|%M)	Diagnosis	Y-BOCS	Treatment	Psychometrics	Task	Results
Janeck et al., [28]	OCD	31	31.9 ± 10.2	39|61	DSM-IV	22.0 ± 6.3	48% medicated	−	Suppression of negative thought	↑overall frequency and distress from negative thought; ↑number of participants with negative thought after suppression.
	Healthy	32	31.2 ± 13.5	66|34		−	−			
Tolin et al., [26]	OCD	15	29.6 ± 9.9	50|50	DSM-IV	23.8 ± 5.4	67% medicated; 73% CBT	−	Suppression of neutral thought	↑frequency of target thought during suppression; ↑frequency and time thinking about target thought overall.
	Healthy	14	26.9 ± 6.5	43|57		−	−			
	OCD	15	25.8 ± 10.1	36|64	DSM-IV	24.2 ± 5.3	75% medicated; 75% CBT	−	Suppression of neutral thought	↓detection time for words related to target thought versus non-related words and non-words during suppression.
	Healthy	13	25.5 ± 6.0	61|39		−	−			
Tolin et al., [27]	OCD	17	29.6 ± 9.9	50|50	DSM-IV	23.8 ± 5.4	67% medicated; 73% CBT	−	Suppression of neutral thought	↑frequency of target thought during suppression; ↑internal meaning (weakness/uncontrollable thoughts) of suppression failure.
	Healthy	8	25.1 ± 4.8	37|63		−	−			
Najmi et al., [29]	OCD	20	29.0 ± 12.0	55|45	DSM-IV	obsessions 11.1 ± 3.1; compulsions 10.7 ± 4.7	95% medicated	−	Suppression, focused distraction, or acceptance of intrusive thoughts	↑distress during all conditions; ↑intrusive thoughts after and during suppression; ↑distress after versus during suppression; ↑distress after suppression versus focused distraction and acceptance; ↑intrusive thoughts after suppression versus acceptance; ↓distress after versus during acceptance.
	Healthy	20	30.0 ± 9.0	65|35		obsessions 1.5 ± 2.0; compulsions 1.0 ± 1.7	−			
Fink et al., [30]	OCD contamination/cleaning	30	33.3 ± 11.4	59|41	DSM-IV	23.0 ± 6.1	60% medicated	↓ERQ reappraisal and ↑ERQ suppression	Mental imagery rescripting or cognitive reappraisal of disgust-inducing pictures	↑disgust ratings before the task.
	Healthy	30	32.8 ± 11.9	59|41		−	−			

OCD—obsessive-compulsive disorder; F—female; M—male; Y-BOCS—Yale-Brown Obsessive Compulsive Scale; DSM—Diagnostic and Statistical Manual of Mental Disorders; CBT—cognitive-behavioral therapy; ERQ—Emotion Regulation Questionnaire. * Studies with overlapping samples.

**Table 2:** Summary of the studies with behavioral and/or neurobiological measures of cognitive regulation. The psychometrics and results comprise direct statistical comparisons between groups (OCD vs. healthy).

Study	Groups	Size	Age (years)	Gender (%F|%M)	Diagnosis	Y-BOCS	Treatment	Psychometrics	Technique	Task	Behavioral Results	Brain Activity Results
Koçak et al., [33]	OCD	12	27.0 ± 5.8	50|50	DSM-IV	20.2 ± 6.2	66% medicated	−	fMRI	Maintenance, suppression, or manipulation of a mental image.	↑performance score during suppression.	↓activity in R inferior parietal lobe, R posterior cingulate, and R superior frontal gyrus for all conditions.
	Healthy	12	25.1 ± 3.32	50|50		−	−					
Simon et al., [32]	OCD	21	33.1 ± 10.8	62|38	DSM-IV	21.2 ± 6.8	Medication-free; 33% CBT	−	fMRI	Appraisal or distraction of OCD-related, aversive, or neutral pictures.	−	↓activity in L amygdala, L dorsal anterior cingulate cortex, L insula, L postcentral gyrus, and R anterior cerebellum during distraction for OCD-related pictures.
	Healthy	21	33.1 ± 10.1	62|38		−	−					
Paul et al., [31]	OCD	24	31.7 ± 9.1	54|46	DSM-IV	22.2 ± 4.1	37% medicated; 37% CBT	↓ERQ reappraisal; ↓CERQ positive refocusing; ↑CERQ catastrophizing.	EEG	Cognitive reappraisal or cognitive distraction of neutral, aversive, and OCD-related pictures.	↓arousal for aversive pictures after reappraisal compared to distraction.	Unchanged Late Positive Potential amplitude during reappraisal and distraction (↓healthy).
	Healthy	24	31.2 ± 8.2	54|46		−	−					
de Wit et al., [24]; Thorsen et al., [25]	OCD	43	37.6 ± 10.0	51|49	DSM-IV	21.6 ± 6.1	Unmedicated for ≥ 4 weeks	↓ERQ reappraisal.	fMRI	Cognitive reappraisal of fearful and OCD-related pictures.	↑distress reduction during OCD-related reappraisal.	Fear reappraisal: ↓activity in R superior temporal gyrus and L middle frontal gyrus, and ↓functional connectivity in L posterior insula and R amygdala; OCD-related reappraisal: ↑activity in R superior frontal gyrus ad R lingual gyrus (uncorrected results).
	Healthy	38	39.0 ± 11.3	53|47		0.0 ± 0.0	−					
Maria Picó-Pérez et al., [34]	OCD	73	37.7 ± 10.2	41|59	DSM-IV	22.1 ± 6.3	92% medicated	↓ERQ reappraisal and ↑ERQ suppression.	fMRI	−	−	Negative correlation between L amygdala–L posterior insula functional connectivity and reappraisal score in controls but not OCD.
	Healthy	42	39.4 ± 9.8	48|52		−	−					

OCD—obsessive-compulsive disorder; F—female; M—male; Y-BOCS—Yale-Brown Obsessive Compulsive Scale; DSM—Diagnostic and Statistical Manual of Mental Disorders; CBT—cognitive behavioral therapy; ERQ—Emotion Regulation Questionnaire; CERQ—Cognitive Emotion Regulation Questionnaire; fMRI—functional magnetic resonance imaging; EEG—electroencephalography; L—left; R—right.

**Table 3:** Description of the assessment of the quality of the studies included in the systematic review based on the National Institute for Clinical Excellence (NICE) checklist for case-control studies.

Study	Definition of Appropriate and Focused Question	Selection of Cases and Controls from Comparable Populations	Use of Same Exclusion Criteria for Cases and Controls	Participation Rate for Cases and Controls	Comparison of Similarities/Differences between Participants and non-Participants	Definition and Differentiation between Cases and Controls	Clear Definition That Controls Are Not Cases	Selection of Measures to Prevent Knowledge of Primary Exposure from Influencing Case Ascertainment	Standard, Valid, and Reliable Measurement of Exposure Status	Identification and Accountability (Design/Analysis) of Potential Confounders	Description of Confidence Intervals
Janeck et al., [28]	Well covered	Poorly addressed	Adequately addressed	Not addressed	Not addressed	Well covered	Well covered	Not applicable	Not applicable	Not addressed	Well covered
Tolin et al., [26]	Well covered	Poorly addressed	Adequately addressed	Not addressed	Not addressed	Well covered	Well covered	Not applicable	Not applicable	Not addressed	Poorly addressed
Tolin et al., [27]	Well covered	Poorly addressed	Adequately addressed	Not addressed	Not addressed	Well covered	Well covered	Not applicable	Not applicable	Poorly addressed	Poorly addressed
Najmi et al., [29]	Well covered	Well covered	Adequately addressed	Not addressed	Not addressed	Well covered	Well covered	Not applicable	Not applicable	Adequately addressed	Poorly addressed
Koçak et al., [33]	Well covered	Well covered	Adequately addressed	Not addressed	Not addressed	Well covered	Well covered	Not applicable	Not applicable	Not addressed	Poorly addressed
Simon et al., [32]	Well covered	Well covered	Adequately addressed	Not addressed	Not addressed	Well covered	Well covered	Not applicable	Not applicable	Well covered	Adequately addressed
de Wit et al., [24]	Adequately addressed	Well covered	Well covered	Not addressed	Not addressed	Well covered	Well covered	Not applicable	Not applicable	Not addressed	Poorly addressed
Paul et al., [31]	Well covered	Well covered	Adequately addressed	Not addressed	Not addressed	Well covered	Well covered	Not applicable	Not applicable	Well covered	Adequately addressed
Fink et al., [30]	Well covered	Well covered	Adequately addressed	Not addressed	Not addressed	Well covered	Well covered	Not applicable	Not applicable	Not addressed	Adequately addressed
Thorsen et al., [25]	Well covered	Well covered	Well covered	Not addressed	Not addressed	Well covered	Well covered	Not applicable	Not applicable	Well covered	Adequately addressed
Maria Picó-Pérez et al., [34]	Well covered	Well covered	Adequately addressed	Not addressed	Not addressed	Well covered	Well covered	Not applicable	Not applicable	Well covered	Adequately addressed

The NICE checklist involves the following evaluation categories for each item: Well covered; Adequately addressed; Poorly addressed; Not addressed; Not reported; Not applicable.

## Supplementary Materials

The following are available online at https://www.mdpi.com/2076-3425/10/11/797/s1, Table S1: Detailed description of the behavioral tasks of the studies included in the systematic review.

## Abbreviations

OCD	obsessive-compulsive disorder;
vlPFC	ventrolateral prefrontal cortex;
dlPFC	dorsolateral prefrontal cortex;
ACC	anterior cingulate cortex;
Y-BOCS	Yale-Brown Obsessive Compulsive Scale;
DSM	Diagnostic and Statistical Manual of Mental Disorders;
fMRI	functional magnetic resonance imaging;
dmPFC	dorsomedial prefrontal cortex;
CBT	cognitive-behavioral therapy.
