# Supplementary material for: A Systematic Review of Behavioral, Physiological, and Neurobiological Cognitive Regulation Alterations in Obsessive-Compulsive Disorder
Source: Brain Sci. 2020 Oct 29;10(11):797. doi: 10.3390/brainsci10110797 (PMC7692269; doi:10.3390/brainsci10110797)
Supplement: Supplementary file 1 [file brainsci-10-00797-s001.pdf]

Table 1. Detailed description of the behavioral tasks of the studies included in the systematic review.

Study	Task description
Janeck et al. 1999	Press a bell every time a personally relevant intrusive thought (selected before the experiment) occurs in two different conditions: (1) think freely (baseline); (2) try to suppress the thought (suppression).
Tolin et al. 2002b	Experiment 1: Press a computer key every time a neutral thought of a "white bear" occurs in three different conditions: (1) think freely (baseline); (2) try not to think of a "white bear" (suppression; immediate enhancement); (3) think freely again (rebound effect).<br>Experiment 2: Press the computer key "A" every time a word appears (e.g. "house") and the key "L" every time a non-word appears (e.g. "jwose") while holding a 6-digit number in memory, in two different conditions: (1) try not to think of a "white bear" (suppression; immediate enhancement); (2) think freely (rebound effect). Words consisted of a target word ("bear") and non-target words (e.g. "house").
Tolin et al. 2002a	Press a computer key every time a neutral thought of a "white bear" occurs in three different conditions: (1) think freely (baseline); (2) try not to think of a "white bear" (suppression; immediate enhancement); (3) think freely again (rebound effect).
Najmi et al. 2009	Press a clicker every time a personally relevant intrusive thought (selected before the experiment) occurs in six different conditions: (1) think freely (baseline); (2) try not to think of the thought (suppression); (3) try not to think of the thought by focusing on a weekend with friends (focused distraction); (4) watch the thought by imagining that it is "coming out of the ears on little signs held by marching soldiers". Do not argue, avoid, or make the signs go away (acceptance); (5) create associations to the thought by thinking of things related to the thought (creating associations); (6) think freely again.
Koçak et al. 2011	Imagine an abstract shaped image (shown before the experiment on a white paper) in five different conditions: (1) imagine the image of the shape continuously (imagination); (2) suppress the image of the shape by trying to see a blank paper (suppression); (3) erase the shape by tracing the outline (erasing); (4) imagine freely and change the image with other intrusive images (free-imagination); (5) rest (resting).
Simon et al. 2014	Visualize tailored OCD-related pictures and aversive and neutral pictures in two different conditions: (1) press a button if the picture is unpleasant (self-referential evaluation); (2) press a button when the bars in the foreground are parallel (distraction).
Paul et al. 2016	Visualize tailored OCD-related, aversive, and neutral pictures in three different conditions: (1) do not attempt to alter elicited emotions (maintain emotions); (2) generate neutral thoughts/images unrelated to the picture (distraction); (3) change the picture emotional meaning (e.g. think that situation is not real or has different outcomes) (reappraisal).
Fink et al. 2018	Visualize tailored disgust inducing pictures in two different conditions: (1) rescript the picture into a positive picture (rescripting); (2) think of alternative perspectives for the picture (reappraisal). A third control condition consists of watching a video of swimming fishes and count the number of times a yellow fish appears.
de Wit et al. 2015; Thorsen et al. 2019	Visualize OCD-related and fear-inducing pictures in two different conditions: (1) experience the picture naturally (attendance); (2) imagine a more positive outcome/interpretation of the picture or imagine that the situation is not real (reappraisal). Neutral pictures are also presented in the attendance condition.
Maria Picó-Pérez et al. 2019	-

OCD—obsessive-compulsive disorder.
